# Tension Hemothorax in Aortic Rupture: A Case Report

**DOI:** 10.3390/medicina57080790

**Published:** 2021-07-31

**Authors:** Jana Pometlová, Roman Madeja, Jiří Demel, Renata Ječmínková, Václav Procházka, Miroslav Kitka, Leopold Pleva

**Affiliations:** 1 Department of Trauma Surgery, University Hospital Ostrava, 17. listopadu 1790, 708 52 Ostrava, Czech Republic; roman.madeja@fno.cz (R.M.); jiri.demel@fno.cz (J.D.); leopold.pleva@fno.cz (L.P.); 2 Institute of Medicine of Disasters, Faculty of Medicine, University of Ostrava, Syllabova 19, 703 00 Ostrava, Czech Republic; 3 Accident&Emergency Department, University Hospital Ostrava, 17. listopadu 1790, 708 52 Ostrava, Czech Republic; renata.jecminkova@fno.cz; 4 Department of Intensive Medicine, Emergency Medicine and Forensic Studies, Faculty of Medicine, University of Ostrava, Syllabova 19, 703 00 Ostrava, Czech Republic; 5 Department of Radiology, University Hospital Ostrava, 17. listopadu 1790, 708 52 Ostrava, Czech Republic; vaclav.prochazka@fno.cz; 6 Clinic of Trauma Surgery, Pavel Jozef Safarik University and Louis Pasteur University Hospital, 040 11 Košice, Czech Republic; miroslav.kitka@upjs.sk

**Keywords:** trauma-induced aortic rupture, blunt thoracic injury, chest drainage, hemothorax

## Abstract

*Background:* The standard ATLS protocol calls for chest drain insertion in patients with hemothorax before performing further diagnostic steps. However, if trauma-induced thoracic aortic rupture is the underlying cause, such drainage can lead to massive bleeding and death of the patient. *Case report:* This report describes a case of a polytrauma patient (car accident), aged 21, with symmetrical chest and decreased breath sounds dorsally on the left. An urgent CT scan revealed subadventitial Grade III thoracic aortic transection with mediastinal hematoma, a massive left-sided hemothorax with mediastinal shift to the right, and other injuries. Stent-graft implantation with subsequent left hemithorax drainage was urgently performed, during which the patient became increasingly unstable from the circulatory point of view. This traumatic hemorrhagic shock was successfully managed at the ICU. *Conclusion:* Although hemothorax is a serious condition requiring rapid treatment, the knowledge of its origin is of utmost importance; performing chest drainage without bleeding control can lead to circulatory instability and death of the patient. Hence, where aortic injury can be suspected based on the mechanism of the injury, it is beneficial to perform spiral CT angiography for accurate diagnosis first and, in cases of aortic injury, to control the bleeding prior to drainage.

## 1. Introduction

A thoracic aortic injury occurs in approx. 2% of patients with blunt thoracic injury and usually represents a life-threatening condition [1,2]. Thoracic aortic rupture resulting from blunt injury leads to on-site death in 75% to 90% of cases [3,4,5,6] and is the cause of up to 18% of deaths in car accidents [3,7]. Only 10–20% of patients with trauma-induced aortic rupture survive until hospital admission [3,4,8,9,10,11]. Unless immediately diagnosed and treated, up to 30% of patients admitted (to hospital) die within 6 h of admission [8,12]. However, appropriate surgical and endovascular treatment can significantly reduce the death rate [1,13,14,15,16]. In this paper, we report a case of a patient with left-sided hemothorax caused by an aortic rupture who was treated by emergent stent-graft placement and subsequent left chest drainage.

## 2. Case Presentation

**Figure 1:**
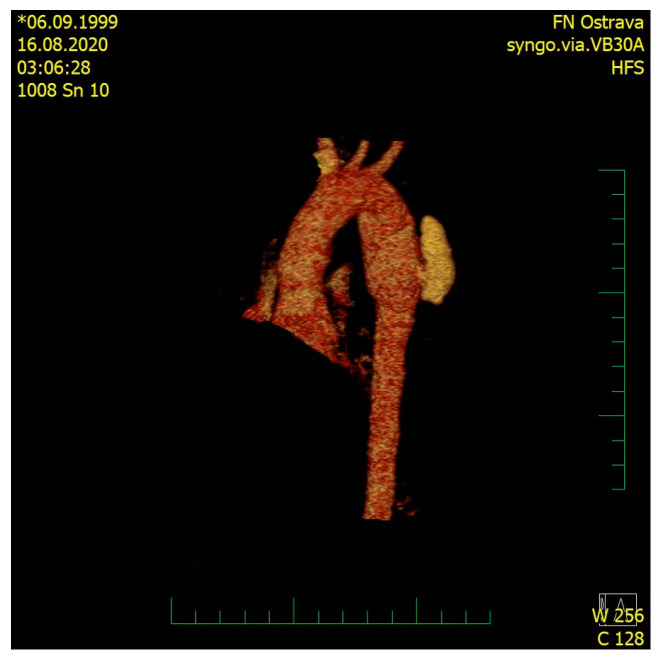
CT 3D reconstruction of the aortic arch with the leak of the contrast agent.

**Figure 2:**
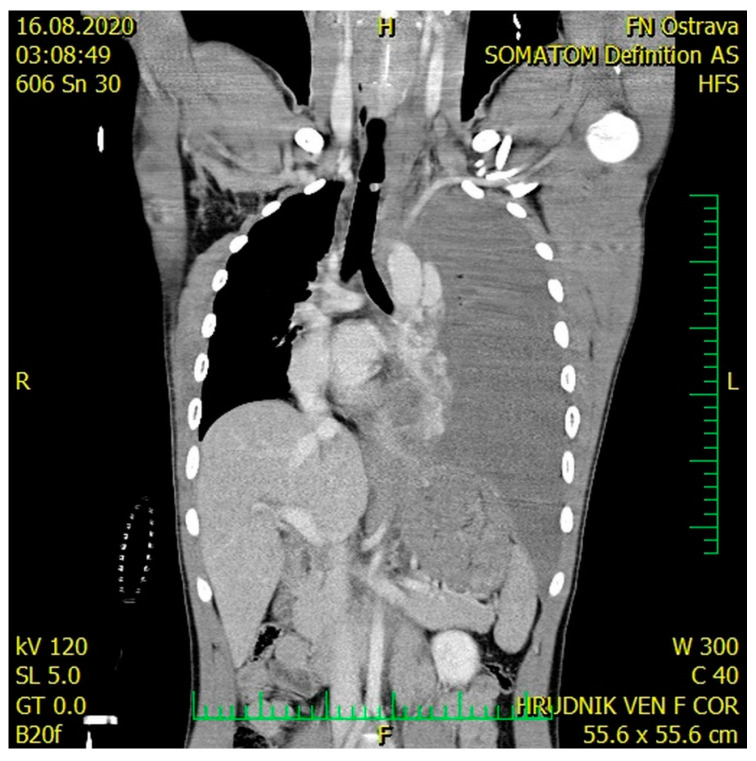
CT scan—large left-sided tension hemothorax with the mediastinal shift.

A male, 21 years old, a victim of a car accident, was brought to the Accident and Emergency (A&E) Department of the University Hospital Ostrava. On the site of the accident, the patient was somnolent and complained of pain in the lower left limb. Airway control at the scene was achieved by orotracheal intubation and the patient was put on ventilation, which continued until Day 2. The left lower limb was stabilized using a vacuum splint. Upon admission to the hospital, the blood pressure was 50/20, pulse 140/min, oxygen saturation 95%. Clinical examination revealed a symmetrical chest, left-sided decreased breath sounds on auscultation, the abdomen was soft, and there was a pathological movement of the left lower limb. The therapy of the hemorrhagic shock initiated during transport was continued, and two whole blood unit transfusions and three units of red blood cells were applied (life-saving indication) along with vasopressor support and IV fluids. After stabilizing the patient, an urgent contrast CT scan was performed, in which Grade III aortic thoracic transection—according to the simplified Vancouver classification—with subadventitial leak of the contrast agent was detected in the mid descending thoracic aorta, along with mediastinal hematoma, massive left-sided hemothorax with mediastinal shift to the right, contusion–laceration injuries to the right lower lobe, minor fissures in the spleen and liver, hematoma in the liver region without any signs of leaks of the contrast agent, and fissures in both kidneys without leak of the contrast agent (Figure 1 and Figure 2). The X-ray revealed a comminuted fracture of the left proximal femur.

**Figure 3:**
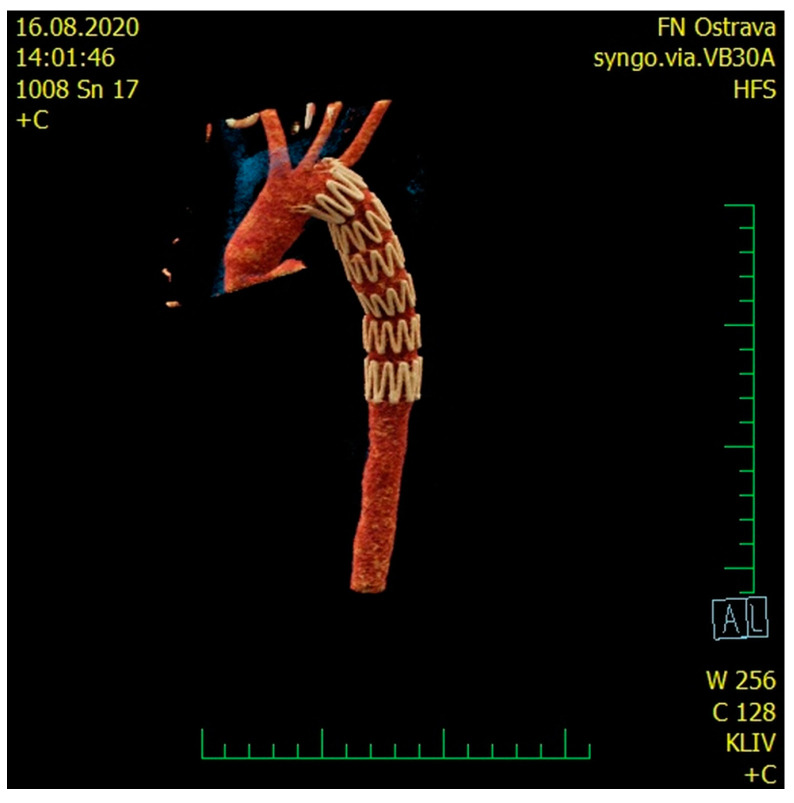
CT 3D reconstruction of the aortic arch with the implanted aortic stent-graft.

A stent-graft was urgently placed (under continuing general anesthesia) into the injured thoracic aorta (Figure 3). A Zenith ZDE6 24/117 stent-graft was implanted, not covering the left subclavian artery. Subsequently, drainage of the left hemithorax was performed. After the drain insertion, 3000 mL of sanguinolent content was evacuated; during the evacuation, however, the patient became increasingly unstable, resulting in circulatory arrest; after a 10 min long resuscitation, circulation was re-established.

The femoral fracture was primarily stabilized by extension and the traumatic–hemorrhagic shock was gradually eliminated during the subsequent intensive care. The next day, once the patient was stabilized, osteosynthesis of the femoral fracture using the long femoral nail was performed. The injuries to the liver, spleen and kidneys were Grade I; therefore, these injuries were treated conservatively.

The patient’s condition gradually improved during subsequent hospitalization. Follow-up CT examination demonstrated that the thoracic endograft was well placed, distal to the left subclavian with complete exclusion of the traumatic pseudoaneurysm and no endoleak; the chest drainage was visible apically on the left, along with a persisting 20 mm dorsobasal left-sided fluidothorax and without hemoperitoneum. Post-contusion changes were apparent on the left lung base; the spleen and liver showed no post-contusion changes. The chest drain was removed on Day 10 after the injury, and on Day 14 after the injury, the patient was discharged to the outpatient care unit.

## 3. Discussion

Traumatic injury to the thoracic aorta is mainly caused by a deceleration mechanism as a result of falls from heights over 3 m, impacts at speeds over 50 km/h, in drivers who did not use seat belts, in passengers catapulted from a vehicle, or in pedestrians struck by a car. In a sudden change of speed, the descending aorta remains fixed in the dorsal mediastinum while the heart and the ascending aorta move forward by inertia. This motion causes the rupture of the aorta in the region of the isthmus [4,7,17]. Less commonly, the aortic injury can arise during blunt trauma through pulling as a result of dislocated fractures of thoracic vertebrae or high pressure acting at the moment of the impact on the non-compressible blood in the aorta [17,18,19]. Fissures in the aortic wall are most commonly transverse and can affect the entire perimeter of the aorta, or just one part. The intima is injured first, followed by the media and adventitia. In patients with aortic trauma, the intima and media are most commonly injured (60% of cases) [17,20]. Complete transection of the aorta is less common, although it causes greater blood loss and is more lethal if it occurs. Mediastinal hematoma can tamponade the bleeding and contain the aortic rupture, which may increase the chances of survival [4,8,17].

Clinical symptoms of the thoracic aortic rupture are well known [17,21]. Clinical injuries to the aorta are, however, often difficult to diagnose in polytrauma patients because they can be masked by concomitant injuries. This was also true in our patient, who had hypotension without obvious signs of bleeding.

Chest X-ray is one of the basic examination methods in trauma patients [17,18]. According to ATLS guidelines, the chest X-ray is considered part of the primary examination of trauma patients. Aortic rupture, however, does not clearly show on the X-ray; it manifests by widening of the mediastinum and change in the aortic contour in mediastinal hematoma and aortic arch pseudoaneurysm. If the mediastinal hematoma communicates to the hemothorax, the chest X-ray shows the signs of a large hemothorax. Nowadays, a spiral contrast CT angiography is a standard part of the examination of patients with high-energy trauma, which has good sensitivity in detecting aortic injuries [17] and facilitates a quick choice of a suitable treatment approach.

The clinical presentation of patients with thoracic aortic rupture can vary depending on the severity of the rupture. Smaller ruptures may be virtually undetectable by simple physical examination and revealed only on CT angiography. This emphasizes the importance of CT angiography, because the clinical presentation in such cases can indeed be extremely non-specific, manifesting only as chest pain, which is expected after high-energy accidents. On the other hand, where massive hemothorax is present, this may also be caused by reasons other than aortic rupture, such as any other thoracic or pulmonary vessels. Clinical signs can also be similar to those found, e.g., in tension pneumothorax or cardiac tamponade. Again, spiral CT angiography can be instrumental in distinguishing these injuries.

Traumatic aortic rupture can be treated surgically using extracorporeal circulation and left-sided thoracotomy or, as is increasingly the case, using endovascular stent-graft placement. Despite advances in surgical and resuscitation techniques, mortality and morbidity of the surgical approach remain high, with a risk of death of 5–31% and paraplegia 0.7–8.7% [8,9,22,23]. Most deaths occur in the postoperative period, often related to other injuries (intracranial hematoma, abdominal bleeding, or unstable spine with spinal cord injury) [8,24]. The surgical approach necessitating extracorporeal circulation and anticoagulation treatment is contraindicated in central nervous system trauma, myocardial or pulmonary contusion, and in trauma-induced coagulopathy; at least one of these is, however, usually present in most polytrauma patients [8,21,25].

Endovascular treatment (i.e., thoracic endovascular aortic repair, TEVAR) by stent-graft placement is associated with a lower hospital mortality rate than open surgical therapy [8,26,27]. Similarly, from the perspective of morbidity, endovascular treatment is also associated with the lower occurrence of complications compared to open surgical therapy [8,11,28]. For these reasons, the endovascular approach become the standard of care has over the last few decades.

According to ATLS guidelines, chest drainage is indicated when hemothorax is detected by X-ray; depending on the amount of evacuated blood and of persisting blood loss by a chest tube, surgical revision may be indicated to control the source of the bleeding. In aortic injuries, however, the chest drainage or thoracotomy can reduce the tamponading effect of the mediastinal hematoma/hemothorax on the source of bleeding, which may lead to the aggravation of bleeding from the injured aorta. For this reason, urgent surgical treatment is burdened with high mortality if performed without the knowledge of the bleeding source or, at least, exclusion of aortic injury.

In polytrauma patients with hemothorax and chest drainage, it is necessary to compensate for the evacuated blood with intravenous fluid resuscitation in appropriate amounts and time scale. (ATLS) Evacuation of more than 1–1.5 L of fluids in under 30 min leads to fast changes in the pressure in the thoracic cavity, which can cause problems with patient’s hemodynamics, a rapid re-expansion of the pulmonary parenchyma, and then can lead to the development of a pulmonary edema [29]. For these reasons, it is necessary to perform the evacuation of the hemothorax gradually and to meticulously monitor the vital functions; this is particularly true in polytrauma patients in critical condition with signs of tension hemothorax.

There is one more point to consider in polytrauma patients—their intubation. It is known that intubation can cause hemodynamic instability; on the other hand, patients with aortic injury are typically unconscious on arrival of the ambulance or suffer with severe and very painful injuries. For these reasons, in countries where an ambulance is always accompanied by a doctor capable of inducing general anesthesia, such patients are almost always intubated on site to prevent aspiration if they are unconscious or to relieve the pain and prevent/reduce shock. Of course, the induction to general anesthesia should be performed in view of preventing hemodynamic instability (for example, employing intravenous anesthetics such as etomidate/ketamine). In addition, intubation should not be performed needlessly, and if the patient’s condition permits, it should be prevented, the patient transported to hospital and examined without intubation; in such cases, an aortic stent placement would preferably be performed in local anesthesia.

## 4. Conclusions

Early, accurate diagnosis is the key for successful treatment of traumatic aortic rupture. The mechanism of the injury is an extremely important piece of information in diagnosing. Chest X-rays can provide information about bleeding into the thoracic cavity and indicate suspicions of a mediastinal hematoma. Spiral CT angiography can reliably depict the grade and type of aortic injury. Knowledge of the source of bleeding and control of bleeding should, in our opinion, precede the chest drainage, especially in cases where aortic (or other massive) bleeding is suspected. Based on these data, it is possible to set the treatment priorities—in this case, the control of bleeding was achieved first by the placement of a stent-graft in the thoracic aorta, and subsequent, gradual evacuation of the hemothorax by thoracostomy driven by the overall circulatory response of the patient.
